# Production of Potato (*Solanum tuberosum* L.) Seed Tuber under Artificial LED Light Irradiation in Plant Factory

**DOI:** 10.3390/plants10020297

**Published:** 2021-02-04

**Authors:** Md Hafizur Rahman, Md Obyedul Kalam Azad, Md Jahirul Islam, Md Soyel Rana, Kui-hua Li, Young Seok Lim

**Affiliations:** 1 Department of Bio-Health Convergence, Kangwon National University, Chuncheon 24341, Korea; hafizknu94@gmail.com (M.H.R.); azadokalam@gmail.com (M.O.K.A.); jahirulislam213@gmail.com (M.J.I.); soyelrana98@gmail.com (M.S.R.); 2 Physiology and Sugar Chemistry Division, Bangladesh Sugar Crop Research Institute, Ishurdi, Pabna 6620, Bangladesh; 3 Agricultural College of Yanbian University, Yanji 133002, China; likuihua70@126.com

**Keywords:** plant growth, potato tuber yield, photosynthetic pigment, primary metabolites

## Abstract

Plant production in a plant factory is an innovative and smart idea to grow food anytime, anywhere, regardless of the outer environment. However, potato pre-basic seed tuber (PBST) production in a plant factory is a comparatively new initiative. Therefore, the aim of this study was to optimize the artificial LED light spectrum to produce PBST in a plant factory. Two potato varieties such as Golden king (V48) and Chungang (V41) were grown in soil substrate under different combination of artificial LED light combinations (such as red+blue+far-red, red+blue+white, blue+far-red, blue+white, red+far-red, and red+white) maintaining photosynthetic photon flux density (PPFD) of 100 mol m^−2^s^−1^, temperature 23/15 °C (day/night), and relative humidity 70%. The study revealed that, overall, potato plant growth (viz.; plant height, node number, leaf number, leaf length and width, fresh and dry weight) was enhanced by the red+far red light for both potato varieties. The total seed tuber number per plant was higher in red+blue+white light for V48, and red+far-red for V41. The fresh tuber weight was the highest in the red+blue+far-red light for V48 and red+blue+white for V41. The highest accumulated photosynthetic pigment (total Chlorophyll, Chlorophyll a, b and Carotenoid) was observed in red+blue+white light for both varieties. The total carbohydrate content and total sucrose content were higher in red+blue+far red and red +far red light treatment for V48 and V41, respectively. Finally, considering all factors, it is concluded that the red+blue+white light combination is deemed to be appropriate for the potato PBST production in plant factory conditions.

## 1. Introduction

The early generation of pre-basic seed potatoes or potato mini tubers is mostly produced in soilless and substrate culture from in vitro potato plantlets or micro tubers in aseptic culture conditions. Generally, potato seed tubers are produced at high densities in the greenhouse using different substrates and open fields. In the open field condition, potatoes’ growth and productivity depend on soil characteristics and topographic conditions [1]. Nowadays, there are multiple techniques which have been assayed to produce potato mini tubers such as hydroponic systems [2] including deep water culture systems [3], the NFT system, and the ebb and flow system [4].

Plant production in controlled environments/plant factories is a well-established agricultural production system for leafy vegetables, microgreens, fruits, and other crop species [5]. However, potato seed tubers/pre-basic seed tuber production in a plant factory is a comparatively new idea where seed tuber can be produced year-round ignoring the external environment. In a plant factory, the plant food production is transferred to a technically optimized building envelope allowing high productivity and assuring good food quality under regulated growing conditions without seasonal interruptions. To produce plants in a controlled environment, light is crucial because artificial light entirely determines the light condition where sunlight is completely restricted. Light regulates plant growth and development, and it directly influences plant photosynthesis resulting in increased carbohydrate and total biomass and ultimate production [6] 

Plant entire developmental process is controlled by light quality, quantity, and photoperiod. Light also has a significant role in altering the nutrients and quality of germinating seed [7]. In greenhouse production, artificial supplemental light compensates the natural light, but in plant factories, the plant solely depends on artificial light. Moreover, it is possible to regulate the plant productivity and nutritional quality by manipulating the light environment in the closed plant factory [8]. Since the blue and red light profoundly affects plant production apart from nutritional quality, yield, and biomass production [9], many plant species are successfully grown either in the greenhouse or in plant factories using red and blue light [10,11]. It has previously been reported that considering the total light that plants receive from natural sunlight, 90% of the absorbed light is B and R light [12]. Blue and red light increased the nutritional and functional status of lettuce, spinach, radish [11], pepper plants [13], and cucumber [14]. It is reported that yield and quality of crops could be improved by managing the light quality and quantity through regulation at the phytochrome photostationary stage, changing the ratio of active phytochrome to total phytochrome [15], exciting the photoreceptors [16], and exciting the enzymatic activity [17].

The appropriate dose of artificial light is plant species dependent. Hogewoning et al. [16] revealed that the photosynthetic capacity of *C. sativus* leaf is twice higher grown at 7% blue compared with 0% blue with a red light at 100 μmol m^−2^ s^−1^ intensity. Matsuda et al. [17] reported that 30 μmol m^−2^ s^−1^ blue light comparing 150 μmolm^−2^ s^−1^ blue light increased the photosynthesis rate in spinach at a total light intensity of 300 μmolm^−2^ s^−1^. Research has shown that red and blue light is the most dominant light band that drives photosynthesis and stimulates plant signaling, respectively, and accelerates the accumulation of secondary metabolites. It has also been confirmed that red and blue light enhances stomatal conductance activity more than other spectral regions [18] where blue light is more effective than red light to open stomata [19].

On a larger scale, plant factories typically produce more plants per unit area than conventional field cultivation; however, energy consumption is higher in the former. Therefore, customized proprieties for plant production is a strong strategy to improve resource use efficiency [20]. Moreover, many research strategies are being developed to grow plant foods in extreme environments, such as in the arctic and even space farming [21]. Given these backgrounds, the design and development of species-specific factors are crucial in establishing food–energy nexus sustainability. Artificial LED provides a means to control the light intensity according to a precise spectrum with lower energy demand.

Artificial LED lighting can regulate the light quality and quantity, thus enabling the required lighting for a particular crop according to its developmental stage [22]. It is hypothesized that an optimal combination of blue, red, far-red, white light under low light irradiation will improve plant production while reducing energy expenses. Therefore, the objective of this study was to determine the optimal B/R/FR/W light ratios under a low light regime by assessing the pre- basic seed tuber yield of two potato varieties gown in plant factory for year-round production.

## 2. Materials and Methods

### 2.1. Plantlet Production and Growth Conditions

*Solanum tuberosum* L. var. Golden king (V48) and Chungang (V41) were selected for the experiments and were obtained from Professor Young-Seok Lim, breeder and owner of these varieties at Kangwon National University. The potato plantlets were grown in in-vitro conditions under artificial white LED light having photosynthetic photon flux density (PPFD) of 100 µmol m^−2^ s^−1^. The photoperiod, relative humidity (RH), and in vitro growth room temperature were 16/8 (day/night), 70%, and 25 °C, respectively.

**Figure 1:**
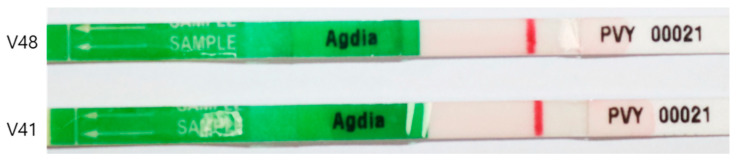
PVY testing results of Golden king (V48) and Chungang (V41) varieties.

Thirty-days-old virus-free tested (ISK 20001/0025, Agdia, Inc., Elkhart, IN, USA) (Figure 1) plantlets were transplanted in commercial substrate soil (Biosangtho. Co., Suncheon, Korea) in a 16-cells seed tray. The newly transplanted plantlets were placed in a greenhouse for three days under shade for acclimatization. After three days, the acclimatized plants were moved to the plant factory equipped with artificial LED light.

### 2.2. Plant Factory and Light-Emitting Diode (LED) Settings

All LED devices used in this study were purchased from ESLEDs Co. Ltd., Seoul, Korea. The artificial red (R, 660 nm), blue (B, 450 nm), far-red (FR, 730 nm), and white (W) LED light were combined to make light treatment according to Table 1. The light intensity was calculated at the plant canopy level, 30 cm from the light panel.

The parameter of the plant factory was as follows: photoperiod 16/8 and 8/16 h (light/dark) at the vegetative growth and at the tuber bulking period, respectively, temperature 25/15 °C (day/night), RH 70%. The day and night temperatures were maintained by the automatic air conditioner. During the study, the potato plants were irrigated with nutrient solution (Table 2) twice a week.

### 2.3. Measurement of Plant Growth Characteristics and Seed Tuber Yield

The potato plant growth characteristics were measured after 40 days of potato growth under artificial LED light in the plant factory. The growth characteristics, such as plant height, leaf number, node number, leaf length and width, fresh weight, and dry weight were measured. The potato pre-basic seed tuber (PBST) was harvested and counted after 90 days of growth when the potato plant was fully matured. The PBST yield was estimated considering the yield of 6 plants/treatment.

### 2.4. Analysis of Photosynthetic Pigment of Potato Plants

The photosynthetic pigments including, chlorophyll a (Chl a), chlorophyll b (Chl b), Total chlorophyll (Chl), and carotenoid of the potato plants were analysed after 40 days of onset of LED light treatment as this time was the peak vegetative stage. The six-plant samples from each treatment were collected for the photosynthetic pigment analysis. The harvested leaves were immediately plunged in liquid nitrogen and then stored at −80 °C for further analysis.

For the determination of photosynthetic pigments, the fresh (3 g) leaves were macerated (10 mL of 80% acetone) using mortar and pestle and placed at room temperature for 15 min. The collected extract was transferred into a tube and centrifuged at 5000 rpm for 10 min. The absorbance was taken at 647, 663, and 470 nm, respectively using a spectrophotometer (UV-1800 240 V, Shimadzu Corporation, Kyoto, Japan). The photosynthetic pigments were determined according to the following formula [23] and expressed as mg/g fresh weight (FW).

Chlorophyll a = 12.25 × A663 − 2.79 × A647

Chlorophyll b = 21.50 × A647 − 5.10 × A663

Total Chlorophyll = 7.15 × A663 + 18.71 × A647

(1) $$\mathrm{Car}=\frac{1000\;\times \;A470-1.82\;\times \;\mathrm{Chl}\;a-85.02\;\times \;\mathrm{Chl}\;b\;}{198}$$

### 2.5. Determination of Total Soluble Carbohydrate (TSC) and Total Soluble Sugar (TSS) Content

The extraction and analysis of TSC and TSS content were determined following the method described by Islam et al. [24]. Briefly, the harvested fresh leave samples (250 mg) were homogenised in 5 mL of ethanol (95%) followed by centrifuging at 5000 rpm for 10 min. After collecting the supernatant, the procedure was repeated with 70% ethanol. Both the supernatants were mixed and kept in a refrigerator (4 °C) for analysis. For analysing the TSC, 0.1 mL of the aliquot was mixed with 1 mL anthrone (200 mg anthrone mixed with 100 mL of 72% sulfuric acid). The mixture was heated at 100 °C for 10 min and then cooled. Total soluble carbohydrate was estimated by using a standard curve of glucose, the detection wavelength was 625 nm, and the results were expressed as mg/g fresh weight. In TSS content, 0.2 mL of the supernatant was mixed with 0.1 mL of KOH (30%) and heated at 100 °C for 10 min. After cooling at room temperature, 3 mL of anthrone (150 mg anthrone mixed with 100 mL 70% sulfuric acid) was added. Ten minutes later, the samples were cooled, and absorbance was read at 620 nm. The TSS concentration was calculated using the standard curve of glucose, and the results were expressed as mg/g fresh weight.

### 2.6. Statistical Analysis

All results were expressed as mean ± SD (standard deviation), and the one-way analysis of variance was performed using Statistix 10 (Tallahassee, FL, USA). Different letters indicate the statistically significant differences between treatments at *p* < 0.05, according to the least significant differences (LSD). The principal component analysis (PCA) was carried out using the OriginLab 10.0 software (OriginLab, Northampton, MA, USA).

## 3. Results

### 3.1. Potato Plant Growth Characteristics and Seed Tuber Yield

**Figure 2:**
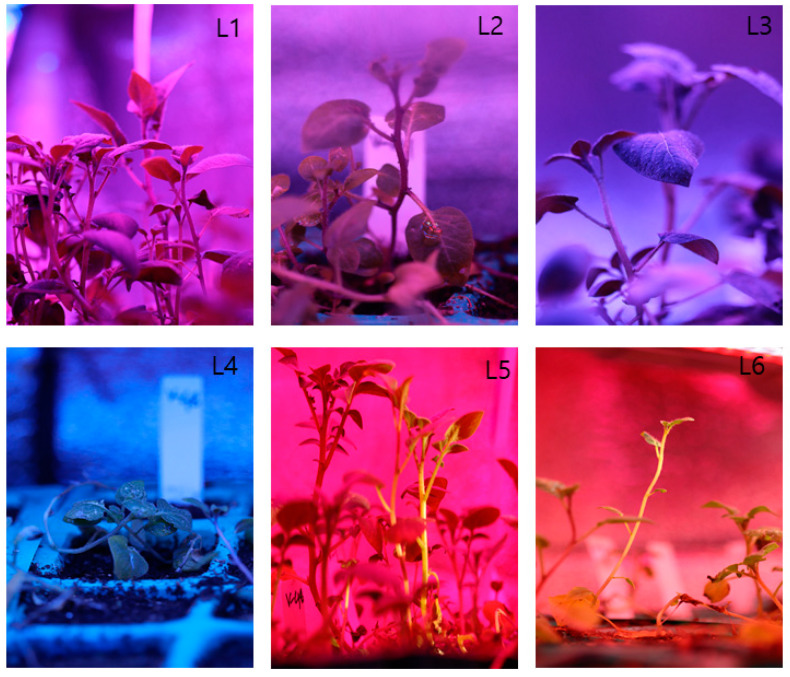
Photographs of potato plants grown under the artificial LED light spectrum. L1 = Red: Blue: Far-red (70:20:10), L2 = Red: Blue: White (70:20:10), L3 = Blue: Far-red (70:30), L4 = Blue: White (70:30), L5 = Red: Far-red (70:30), L6 = Red: White (70:30).

Table 3 indicated the growth characteristics of the potato plants grown under the different LED light spectrum. The study shows that L5 treatment has an overall positive effect on the growth characteristics for both cultivars among the artificial light combination. The plant height, plant fresh, and dry weight was significantly higher in the L5 treatment for both cultivars (Figure 2). The node number was the same within the light combination except L2/L6. The total leaf number was higher in L5 for V48 and in L2 for V41. The leaf length increased in L1/L3/L5 for V48 and L3/L5 for V1.

**Figure 3:**
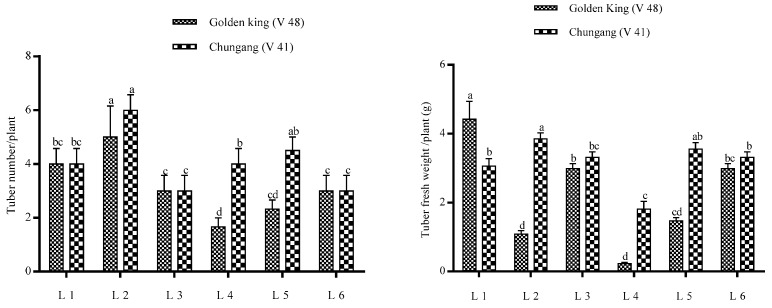

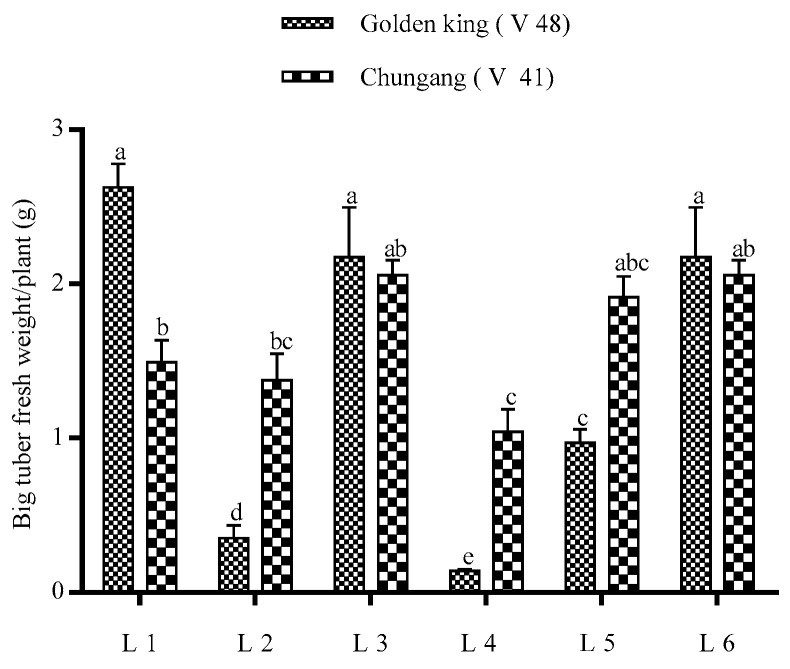
Effects of LEDs light on the yield of potato tubers. Different letters in each bar graph indicate significant differences (*p* < 0.05). Varieties, Golden king (V48) and Chungang (V41). L1 = Red: Blue: Far-red (70:20:10), L2 = Red: Blue: White (70:20:10), L3 = Blue: Far-red (70:30), L4 = Blue: White (70:30), L5 = Red: Far-red (70:30), L6 = Red: White (70:30).

**Figure 4:**
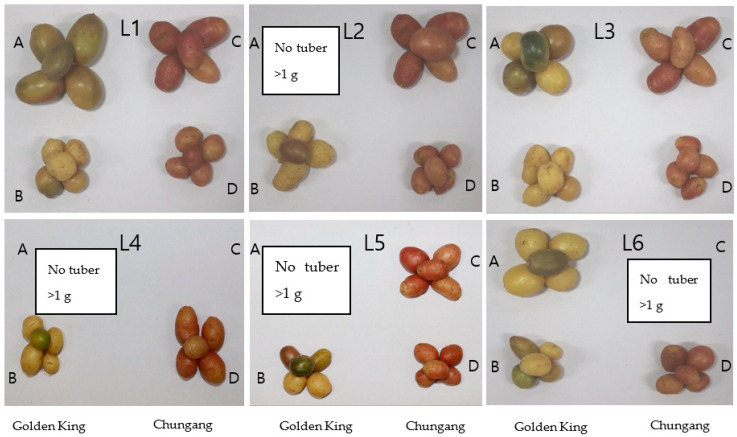
Photographs of potato tubers grown under the artificial LED light spectrum. Golden king (V48): A (tuber weight >1 g); B (tuber weight <1 g). Chungang (V41): C (tuber weight >1 g); D (tuber weight <1 g).

**Figure 5:**
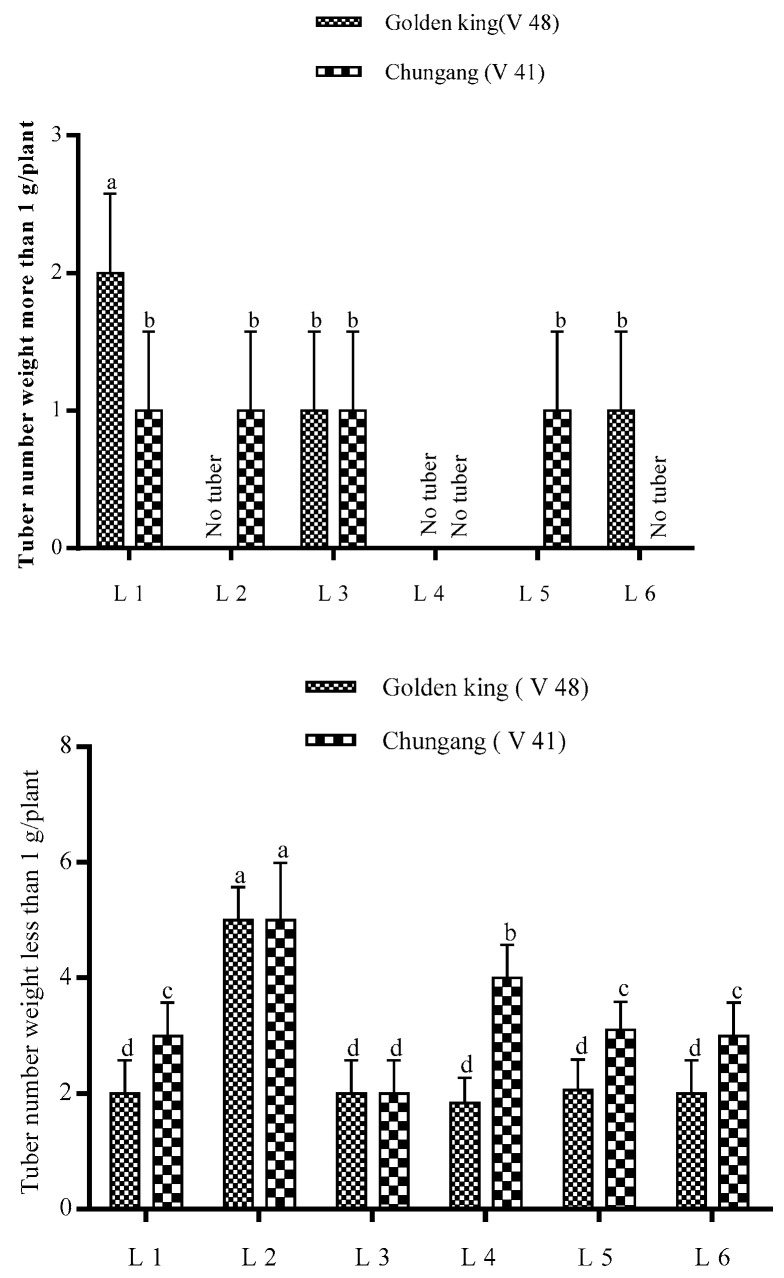
The big tuber fresh weight of potatoes grown under the artificial LED light. Different letters in each bar graph indicate significant differences (*p* < 0.05). Varieties, Golden king (V 48) and Chungang (V 41). L1 = Red: Blue: Far-red (70:20:10), L2 = Red: Blue: White (70:20:10), L3 = Blue: Far-red (70:30), L4 = Blue: White (70:30), L5 = Red: Far-red (70:30), L6 = Red: White (70:30).

The total tuber per plant and tuber fresh weight were demonstrated in Figure 3. It is shown that tuber number per plant was higher in L2 for both cultivars; however, total tuber fresh weight was higher in L1 for V48 and L2 for V41. The comparative bigger tuber’s fresh weight was significantly higher in light treatment L1 in the golden king (V 48), where tuber weight > 1 g was minimal in L4 in both varieties (Figure 4 and Figure 5). On the other hand, L2 and L4 produced very small tubers in size while no tubers were found >1 g.

### 3.2. Analysis of Plant Photosynthetic Pigments and Carbohydrate Content of Potato Plants

The photosynthetic pigments such as total Chlorophyll, Chl a, b, and Carotenoid content of potato plants are shown in Table 4. It is observed that treatment L2 significantly influences the total Chlorophyll, Chl a, b, and total carotenoid content for V48. On the other hand, for V41, total Chlorophyll, Chl a, b and Carotenoid were increased in L1.

**Figure 6:**
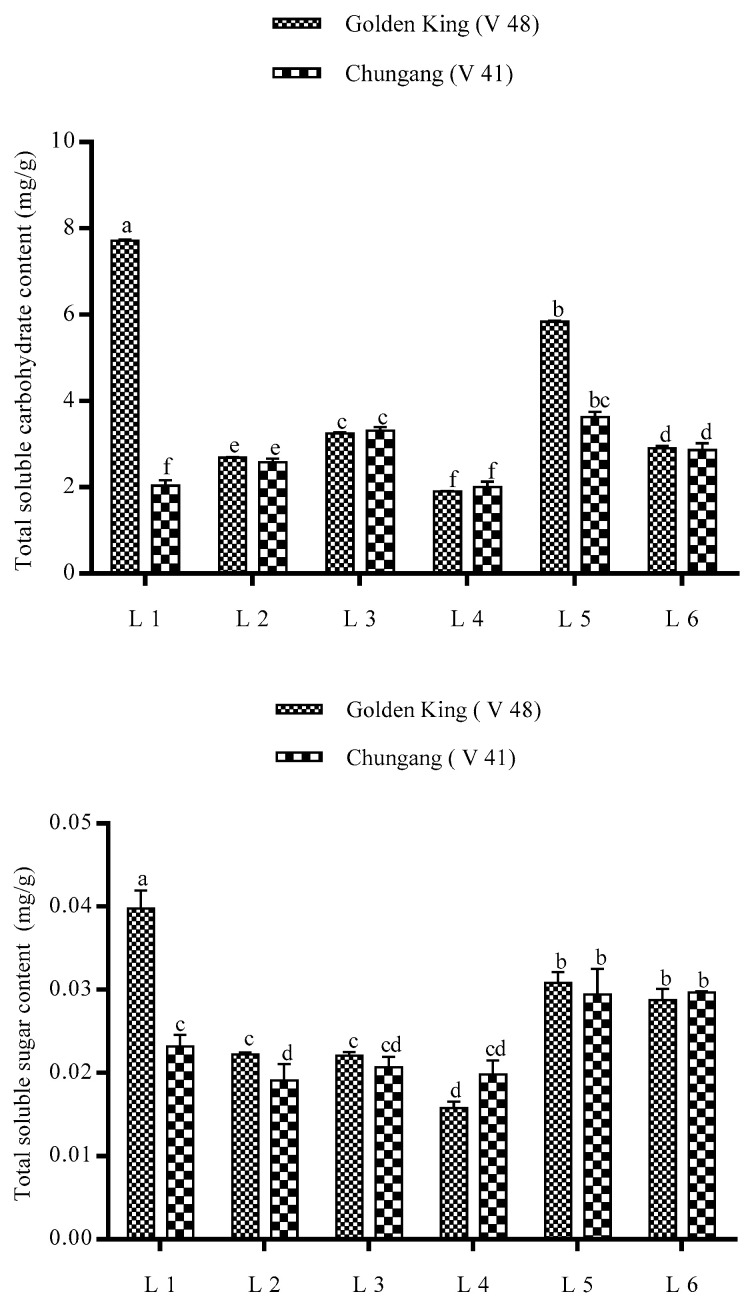
Effect of LEDs light treatment on the total soluble carbohydrate content and total soluble sugar content of potato plants in greenhouse condition. Different letters in each bar graph indicate significant differences (*p* < 0.05). Varieties, Golden king (V48) and Chungang (V41). L1 = Red: Blue: Far-red (70:20:10), L2 = Red: Blue: White (70:20:10), L3 = Blue: Far-red (70:30), L4 = Blue: White (70:30), L5 = Red: Far-red (70:30), L6 = Red: White (70:30).

The total carbohydrate content and total sucrose content, as depicted in Figure 6, was significantly higher in the L1 treatment for V48. However, total carbohydrate and sucrose content was higher in L5 for the V41 cultivar.

## 4. Discussion

### 4.1. Potato Plant Growth Characteristics and Seed Tuber Production

This study demonstrated that the different combination of the artificial LED light spectrum has a significant impact on the growth and seed tuber formation of the potato plants. A common trend observed that the L5 (Red+Far-red) light combination profoundly influences the speedy growth of potato plants (Figure 2). Many studies were published on the optimum dose of the red, blue, and far-red light spectrum for plant growth and development. It is reported that the optimum ratio of red and blue LED light is to be specified and varied to plant species. For instance, the suitable combination of red and blue LED light was 50R/50B for cotton [25], 80R/20B for *Phalaenopsis* and banana [26,27], and 1B/5R for lettuce [20]. It was reported that 65% red + 35% blue light enhanced growth, stem diameter fresh and dry weight of in vitro potato plantlets [28]. The report has demonstrated that potato plantlets that were grown under monochromatic red light were weak and slim with small leaves [29], however, in the current study, potato plants had elongated stems with high biomass when grown under the red +far-red light spectrum.

In our study, the potato plant’s growth was higher at L5 light because of the red and far-red light ratio. Research proved that photosynthetic pigments absorb and convert light energy into chemical energy via complex photosynthetic machinery. Blue and red light play an active role in photosynthesis and stimulate the biosynthesis of carotenoids and chlorophyll [30]. It was also reported that red and far-red light regulates the photosensors that promote the stem elongation while an opposite effect was observed from the blue light [31,32]. These findings are quite similar to our results.

Moreover, it is also proved that blue light activates the stomata and broadens the leaf. It has long been known that blue and red lights induce stomatal opening [33]; however, blue light is more efficient than red [34]. The study has demonstrated that the Red HL and blue regimens induced stomatal opening, while the stomata were more closed under the Red Far-red and white regimens. It seems that neither the red/far-red, nor the blue/red ratio correlated to stomatal movements rather than the low blue/far-red ratio [32]. In our study, L5 treatment plays a crucial role in broadening potato leaves compared to treatment containing blue light. This finding indicated that the light spectrum requirement is plant species related, which supports the previous studies. However, the two potato cultivars (V48, V41) showed a similar pattern of response to the different light spectrum with few exceptions. 

The highest tuber number was obtained in L2 treatment for both varieties; however, total tuber fresh weight was higher in L1 for the V48 variety. It may be due to the smaller tuber size, which increases the number but has less weight. However, total fresh weight was higher in L1 for V48 and L2 for V41. The total tuber fresh weight was decreased in L2, owing to the smaller tuber for V48.

The plant growth was promoted in L5 treatment for both cultivars; however, tuber formation was enhanced in L2 (red+blue+white) treatment. This phenomenon indicated that light requirement for growth and potato tuber formation is independent when grown under artificial LED light in plant factory. There is no evidence of how tuberization is caused by light from various wavelengths [35]. However, the tuber formation of potatoes is directed by the hormonal signal, especially gibberellins (GA) and cytokinin (CK). It is stated that a CK activating enzyme induces de novo homeotic tubers in the potato, which is the phytochromes mediated leaf driving signaling on tuberization [36]. A previous study showed that red light inhibited mini tuber initiation, which is inconsistent with the current research [37]. The effect of regulation on the light spectrum on plant growth can be partly attributed to the regulation of the phytohormone levels in plants [38]. Endogenous hormones in potatoes are affected by light quality [39]. It has been also reported that gibberellic acid (GA3) and abscisic acid (ABA) are closely related to tuber formation and that GA3 inhibits tuber formation whereas ABA promotes it [40,41]. GA3 concentrations decrease in leaves of grape grown under red light [42], and ABA concentrations induced by red light increased in the hypocotyl of cucumber [43]. In the present study, red light facilitated tuber formation, which might be correlated with decreased levels of GA3 and increased ABA concentrations in plants.

It is assumed that this light treatment successfully activated the CK and GA, which induced more tuber. The red light spectrum initiates more biosynthesis of endogenous gibberellin (GA). However, the red:blue (30:70) spectrum generated a static GA level of the plant; consequently, the plant acquired growth signals like stem elongation and stem diameter. As indicated earlier, the combined spectrum of the red and blue light was represented to be further patronizing for stem length compares to white light [44,45,46]. Generally, elongation of the stem depends on cellular proliferation and cell elongation. There was also a significant effect of far-red and, red light on cell numbers and cell length on aspen [47]. It is also reported that the red+blue light spectrum increases the tuber number per potato plant. [28] Overall, the consequence of this study demonstrated that the combined spectrum of red, blue, and white had a significant effect on stem elongation, which ultimately turned into the tuber. Another hormone named indole acetic acid (IAA) is believed to enhance the sink capacity of plant organs [48]. It is reported that red light increases the IAA concentration in potatoes thus, promoting the flow of assimilates into the tubers. The rate of assimilates is also an essential factor for tuber size or weight [49,50]. Enhanced assimilate rates are efficiently partitioned into underground tubers in plant growing under the combined LED blue and red spectrum. This may be the explanation for why most large micro-tubers were found in the combined spectrum of red and blue [49,51].

### 4.2. Analysis of Photosynthetic Pigments and Carbohydrate Content

The photosynthesis pigments such as total chlorophyll, chl a and b, and total carotenoid were significantly increased in L2 treatment (red+blue+white). It is well established that plant pigments receive a particular light spectrum through their light-harvesting antenna, i.e., chlorophyll and carotenoid pigment absorb at wavelengths 400–500 nm and 630–680 nm of the light spectrum [9,52]. Blue light is profusely absorbed by the photosynthetic antenna of plant pigment, which works as a catalytic agent to accumulate the pigment including chlorophyll and carotenoid content in plant leaves [53,54].

It was noted that the red spectrum, combined with the blue spectrum, significantly increases the photosynthetic pigment, including carotenoid compared to other spectrum combinations [49]. A severe malformation of the chloroplast of invitro potato plantlets was found when grown under a monochromatic red light only; however, the red and blue light combination provided more uniform chloroplast in the leaf with increased leaf thickness [28].

Our current study demonstrated that L2 light enhanced the total chlorophyll, chl a, b, and carotenoid content. This finding is consistent with the previous reports of Li et al. [55] and Son et al. [56]. The enhanced chlorophyll and carotenoid content of potato in L2 treatment are due to the stimulation, induction, and synthesis of the principal gene (PAL gene activity) in chlorophyll and carotenoid by red and blue light spectrum [57,58].

The total carbohydrate and sucrose content were significantly increased in L1 treatment Figure 6). Carbohydrates are the final product of photosynthesis and an essential parameter for the plant [9]. Previously, it was reported that red light is the most effective light source for accumulating soluble carbohydrates [55]. The total carbohydrate and sucrose content for both potato cultivars are responded to differently. The treatment L5 induced primary metabolites in potato for V41 however, L1 for V48. It may be due to the genetic variation of the plant, which determines the Calvin cycle and photorespiration and photoprotective mechanisms to effectively utilize light, thus leading to the accumulation pattern of metabolites [59]. Red and blue light has promoted photosynthetic product accumulated in the plants, however, combined blue and red light increase these compounds in the plants, which partially supports our study [60].

Irradiation of blue light significantly increases the soluble-carbohydrate content including, glucose, fructose, sucrose in Chinese bayberry, a similar phenomenon also executed in the strawberry fruit [61]. Previous findings demonstrated that sucrose synthesis is associated with the aggravating activity of Sucrose-phosphate synthase (SPS) gene expression [61], which, combined with red+blue+far-red, have a significant role in increasing soluble carbohydrate and soluble sugar. The higher TSC and TSS content in L1 (Red: Blue: Far-red) treatments also comply with this finding.

### 4.3. PCA Analysis Unveiled the Connections between Variables and Treatments

**Figure 7:**
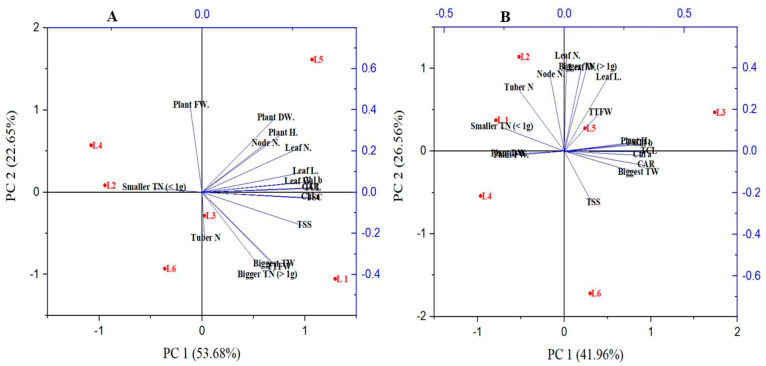
Principal component analysis (PCA) representing patterns and associations between variables and treatments of V48 (**A**) and V41 (**B**).

The PCA analysis was carried out to discover the relationship between different parameters and treatments (Figure 7). The PCA (PC1 and PC2) elements described 76.33% and 68.52% of data variability in V48 and V41. The results demonstrated that most of the plants’ morphological characteristics are associated with the treatment L5 in both genotypes. A higher tuber number is associated with L2 in both the genotypes; moreover, L1 also showed a significant tuber number in both varieties and Chungang variety in L5 respectively. However, with minimal yield performance, tuber number and tuber fresh weight was recorded in L4 in the specific variety Golden king (V48).

However, the L1 and L2 have a parallel relationship with bigger and smaller tuber sizes, respectively. The results also demonstrated that the TSC and TSS of potato plants have a close relationship with the production of tuber size.

In PCA, the lines starting from the central point of the biplots present negative or positive associations of different variables, and their proximity specifies the degree of correlation with a specific treatment (Figure 7). L1, R:B:FR; L2, R:B:W; L3, B:FR; L4, B:W; L5, R:FR; L6, R:W. Plant H., Plant Height; Plant FW., Plant Fresh Weight; Plant DW., Plant Dry Weight; leaf N., Leaf Number; Node N., Node Number; Leaf N., Leaf Number; Leaf L., Leaf Length; Leaf W., Leaf width; Chl a, Chlorophyll a; Chl b, Chlorophyll b; TCL, Total Chlorophyll; TSC, Total Soluble Carbohydrate; TSS, Total Soluble Sugar; Tuber N., Tuber Number; TTFW, Total Tuber Fresh Weight; Biggest TW., Biggest tuber weight; Bigger TN > 1 g, Bigger tuber weight more than 1 g; smaller TN < 1 g, smaller tuber number less than 1 g.

## 5. Conclusions

The current study obtained that potato plant growth and tuber formation are an independent phenomenon, and their response to the artificial light is unique when grown in a plant factory. Red and far-red light spectrum boosted up the growth characteristics; however, red+blue+far-red/white light combination influenced the tuber formation and accumulation of primary metabolites. These results are the preliminary findings to produce seed tuber in a plant factory under artificial light, which provides a fundamental ground to design further artificial LED lighting environment to grow potato PBST in a plant factory.

## Figures and Tables

**Table 1:** Light spectrum combinations and ratios.

Spectrum Combinations	Intensity Ratio (%)	Intensity (µmol m^−2^ s^−1^)	Code Name
R:B:FR	70:20:10	100	L1
R:B:W	70:20:10	100	L2
B:FR	70:30	100	L3
B:W	70:30	100	L4
R:FR	70:30	100	L5
R:W	70:30	100	L6

**Table 2:** Nutrient solution formulations.

Chemical Name	Vegetative Growth Period (Transplantation to 40th Day)		Tuber Bulking Period (41th Days to Harvesting Day)	
	A Tank (50 L)	B Tank (50 L)	A Tank (50 L)	B Tank (50 L)
Ca(NO_3_)	1.5 kg		7.66 kg	
KNO_3_	3.79 kg	3.79 kg	3.54 kg	3.54 kg
(NH_4_)_2_HPO_4_		1.6 kg		1.52 kg
MgSO_4_		4.3 kg		3.68 kg
K_2_SO_4_				1.3 kg
Fe-EDTA	460 g		460 g	30.8 g
MnSO_4_		30.8 g		
H_3_BO_3_		57.2 g		57.2 g
ZnSO_4_		3.6 g		3.6 g
CuSO_4_		1.3 g		1.3 g
(NH_4_)_6_Mo_7_O_24_·4H_2_O		0.4 g		0.4 g

**Table 3:** Effect of artificial LED on the growth parameters of potato plants in plant factory.

Treatments	Plant Height (cm)		Node Number		Leaf Number		Leaf Length (cm)		Leaf Width (cm)		Fresh Weight/Plant (g)		Dry Weight/Plant (g)	
	V48	V41	V48	V41	V48	V41	V48	V41	V48	V41	V48	V41	V48	V41
L1	17.33 ± 3.3 bc	17.01 ± 3.2 c	15.33 ± 2.8 a	13.66 ± 1.4 b	9.61 ± 1.6 ab	9 ± 0.8 ab	5.5 ± 0.4 a	3 ± 0.4 b	2.5 ± 0.4 a	1.5 ± 0.2 b	2.16 ± 0.3 cd	3.35 ± 0.2 d	0.21 ± 0.0 c	0.24 ± 0.0 bc
L2	13 ± 0.8 c	15 ± 0.8 c	12 ± 0.8 ab	14.22 ± 2 a	7.31 ± 1.2 b	9.66 ± 1.2 a	3.03 ± 0.2 b	3.1 ± 0.7 b	1.9 ± 0.2 ab	1.6 ± 0.3 abc	7.18 ± 1.0 ab	4.54 ± 0.3 c	0.4 ± 0.0 b	0.25 ± 0.0 bc
L3	21.6 ± 1.2 b	26 ± 1.6 ab	15.67 ± 1.2 a	14.34 ± 2.9 a	9 ± 0.8 ab	9 ± 0.8 ab	5.4 ± 0.53 a	4.43 ± 0.4 a	2.5 ± 0.1 a	1.86 ± 0.1 a	1.714 ± 0.2 d	0.42 ± 0.0 e	0.17 ± 0.0 c	0.08 ± 0.0 d
L4	14 ± 0.8 c	15 ± 1.6 c	15.31 ± 1.6 a	14.24 ± 2.4 a	7.32 ± 1.6 ab	8 ± 0.8 ab	2.73 ± 0.2 b	1.96 ± 0.1 c	1.55 ± 0.1 b	1.43 ± 0.2 bc	6.25 ± 0.9 b	5.45 ± 0.7 b	0.15 ± 0.0 c	0.19 ± 0.0 c
L5	30.3 ± 1.1 a	27 ± 1.4 a	16.33 ± 1.2 a	14.16 ± 1.2 a	10.63 ± 1.1 a	9 ± 0.8 ab	5.9 ± 0.2 a	4.23 ± 0.5 a	2.5 ± 0.4 a	1.73 ± 0.5 a	9.98 ± 1.7 a	6.91 ± 0.2 a	1.23 ± 0.0 c	0.35 ± 0.0 a
L6	16.31 ± 2.0 bc	19 ± 2.9 bc	11.36 ± 0.8 ab	14.74 ± 0.8 a	6 ± 0.82 b	7.33 ± 1.2 b	2.66 ± 0.4 b	1.13 ± 0.1 c	1.4 ± 0.2 c	0.6 ± 0.1 c	4.44 ± 0.2 c	3.55 ± 0.3 d	0.12 ± 0.0 c	0.27 ± 0.0 b
	df	mss	mss		mss		mss		mss		mss		mss	
Variety	1	11.1 ***	38.02 ***		1.00 ***		13.56 ***		3.67 ***		14.07 ***		0.35 ***	
Treatment	5	199.13 ***	19.36 ^ns^		8.13 ***		10.85 ***		1.06 ***		41.05 ***		0.33 ***	
Variety X Treatment	5	10.37 ***	12.29 ***		3.06 ***		1.15 ***		0.2 ***		3.46 ***		0.21 **	
LSD (0.05)	7.39	7.85	5.61	4.44	3.89	2.19	1.64	0.86	1.25	0.62	3.53	0.75	0.19	0.07

The values are treatment means ± standard deviations. Different letters indicate a significant difference between treatments in the same columns. LSD: least significant difference, df: degree of freedom; mss: mean sum of square; 2 way ANOVA was conducted at *p* ≤ 0.001 (***), 0.01 (**), Varieties: Golden king (V 48) and Chungang (V41). L1 = Red: Blue: Far-red (70:20:10), L2 = Red: Blue: White (70:20:10), L3 = Blue: Far-red (70:30), L4 = Blue: White (70:30), L5 = Red: Far-red (70:30), L6 = Red: White (70:30).

**Table 4:** Effect of LEDs light treatments on photosynthetic pigments, total chlorophyll, and carotenoids.

Treatments	Chlorophyll a (mg/g)		Chlorophyll b (mg/g)		Total Chlorophyll (mg/g)		Carotenoid (mg/g)	
	V48	V41	V48	V41	V48	V41	V48	V41
L1	1.12 ± 0.05 a	0.61 ± 0.05 e	0.74 ± 0.05 b	0.44 ± 0.03 d	2.07 ± 0.11 a	1.06 ± 0.08 d	2.01 ± 0.07 a	1.12 ± 0.05 b
L2	1.76 ± 0.06 a	1.82 ± 0.05 a	1.01 ± 0.06 a	0.95 ± 0.04 a	2.25 ± 0.12 a	1.97 ± 0.02 a	2.19 ± 0.05 a	1.74 ± 0.07 a
L3	0.75 ± 0.05 d	0.99 ± 0.07 a	0.55 ± 0.07c	0.76 ± 0.03 a	1.3 ± 0.12 d	1.46 ± 0.11 b	1.21 ± 0.05 c	0.73 ± 0.04 cd
L4	0.66 ± 0.02 de	0.7 ± 0.04 de	0.45 ± 0.04 d	0.51 ± 0.06 c	1.12 ± 0.05 de	1.22 ± 0.02 cd	1.12 ± 0.06 d	0.69 ± 0.06 d
L5	1.06 ± 0.06 b	0.77 ± 0.03 bc	1.03 ± 0.05 a	0.51 ± 0.05 c	2.04 ± 0.02 a	1.29 ± 0.07 c	1.99 ± 0.07 a	1.04 ± 0.1 b
L6	0.87 ± 0.04 c	0.87 ± 0.03 b	0.69 ± 0.02 b	0.57 ± 0.03 b	1.56 ± 0.02 b	1.44 ± 0.07 b	1.51 ± 0.07 b	0.86 ± 0.06 c
LSD (0.05)	0.1	0.1	0.12	0.03	0.13	0.09	0.13	0.14

The values are treatment means ± standard deviations. Different letters indicate a significant difference between treatments in the same columns. LSD, least significant difference, *p* ≤ 0.05. Varieties, Golden king (V 48) and Chungang (V 41). L1 = Red: Blue: Far-red (70:20:10), L2 = Red: Blue: White (70:20:10), L3 = Blue: Far-red (70:30), L4 = Blue: White (70:30), L5 = Red: Far-red (70:30), L6 = Red: White (70:30).

## Data Availability

Not applicable.
